# Precipitation behavior in a nitride-strengthened martensitic heat resistant steel during hot deformation

**DOI:** 10.1016/j.dib.2015.06.018

**Published:** 2015-07-07

**Authors:** Wenfeng Zhang, Qingyong Su, Mi Xu, Wei Yan

**Affiliations:** a Guilin University of Aerospace Technology, Guilin 541004, China; b Institute of Metal Research, Chinese Academy of Sciences, Shenyang 110016, China

## Abstract

The stress relaxation curves for three different hot deformation processes in the temperature range of 750–1000 °C were studied to develop an understanding of the precipitation behavior in a nitride-strengthened martensitic heat resistant steel (Zhang et al., Mater. Sci. Eng. A, 2015) [1]. This data article provides supporting data and detailed information on how to accurately analysis the stress relaxation data. The statistical analysis of the stress peak curves, including the number of peaks, the intensity of the peaks and the integral value of the pumps, was carried out. Meanwhile, the XRD energy spectrum data was also calculated in terms of lattice distortion.

**Table:** Specifications Table

Subject area	*Material science and engineering*
More specific subject area	*Microstructure, precipitates*
Type of data	*Table, image (x-ray, microscopy, curve),.opj file*
How data was acquired	*Microstructure observation using SEM, TEM, and OM, calculation using XRD, EDAX, Origin software. Raw data gained by Gleeble3500*
Data format	*Raw stress curves data, and analyzed computational data, and raw.jpg files for microstructure*
Experimental factors	*cast*
Experimental features	*Heated at1200* *°C for 5* *min then cooled down to a certain temperature then deformed and isothermal holding for a certain time*
Data source location	*2nd Jinji Road, Qixing District, Guilin, Guangxi, China*
Data accessibility	*data available in this article*

**Value of the data**

• The methods behind the data presented here for particle precipitation behavior during hot deformation of heat resistant steel might be useful for some other kind of alloy.
• The data presented here may facilitate the improvement of the particle nature steel׳s final state.
• The microstructure illustrated in this paper may illuminate softening mechanisms taking place as well as insight into microstructure evolution during hot deformation.

## 1 Data, experimental design, materials and methods

The stress relaxation curve shown here was statistically analyzed in terms of the peak number, the peak intensity and the integral value of the pumps. The XRD energy spectrum was proceed by using the internal standard method (ISTD) through Jade software. Details on these data and analyses are presented below.

## 2 Analysis of the stress relaxation curve

**Fig. 1:**
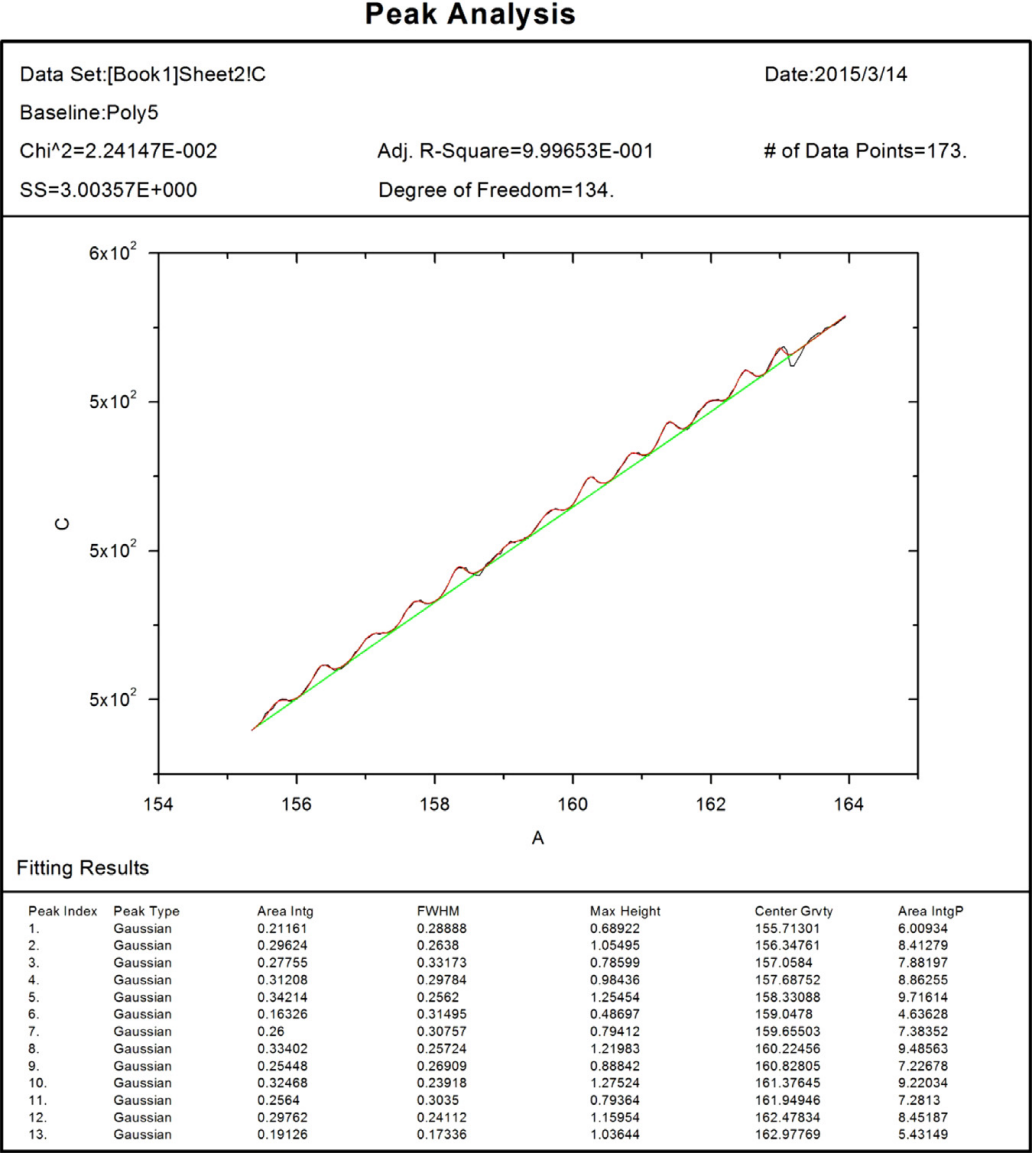
The stress wave statistical analysis.

Two types of stress serrations were shown in the stress curve, including stress wave and sharp stress jump. The first type [1,2] was detailed studied by origin software. First the data of stress and time was imported into the software, and then abstract the interested part of the data and draw out the stress–time curve. A baseline should be created through the function of “create baseline” in “peak analyzer”. By this, the baseline data would be created and output in a new column. Based on this, the baseline data could be subtracted from the stress curve. To do this, the function of “fit peaks (pro)” would be employed in the “peak analyzer” menu. However, during this process, the baseline data location would be assigned. The data of peaks and fitting data would all be outputted in the worksheet. The integrated picture is shown in Fig. 1.

## 3 Analysis of the XRD results

**Fig. 2:**
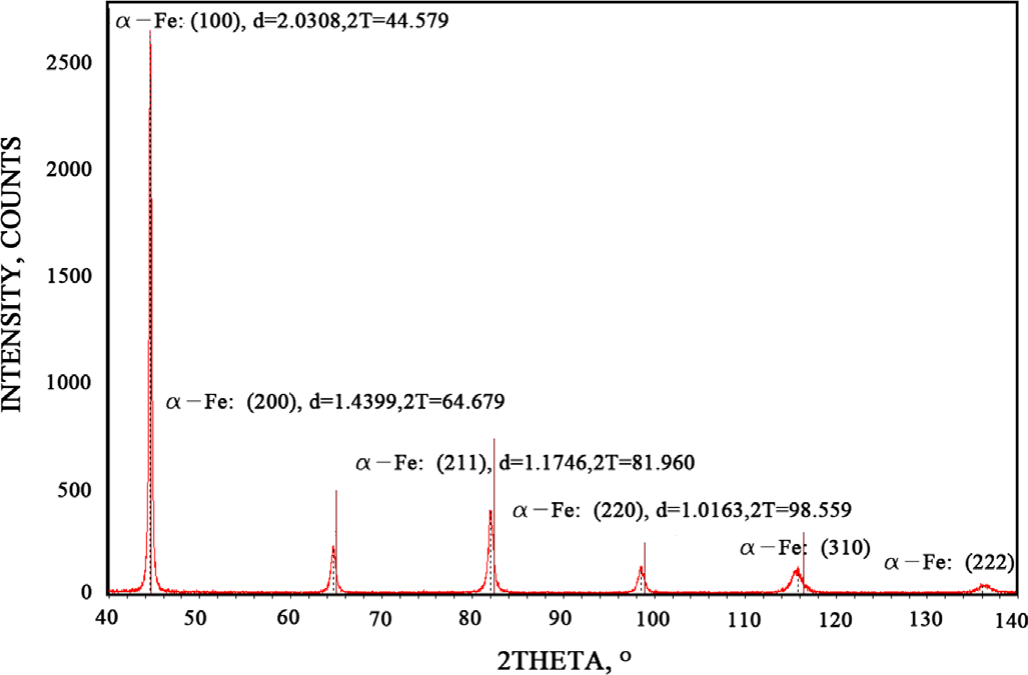
The spectrum of the sample deformed at 940 °C with the 2theta value of 40–140.

**Fig. 3:**
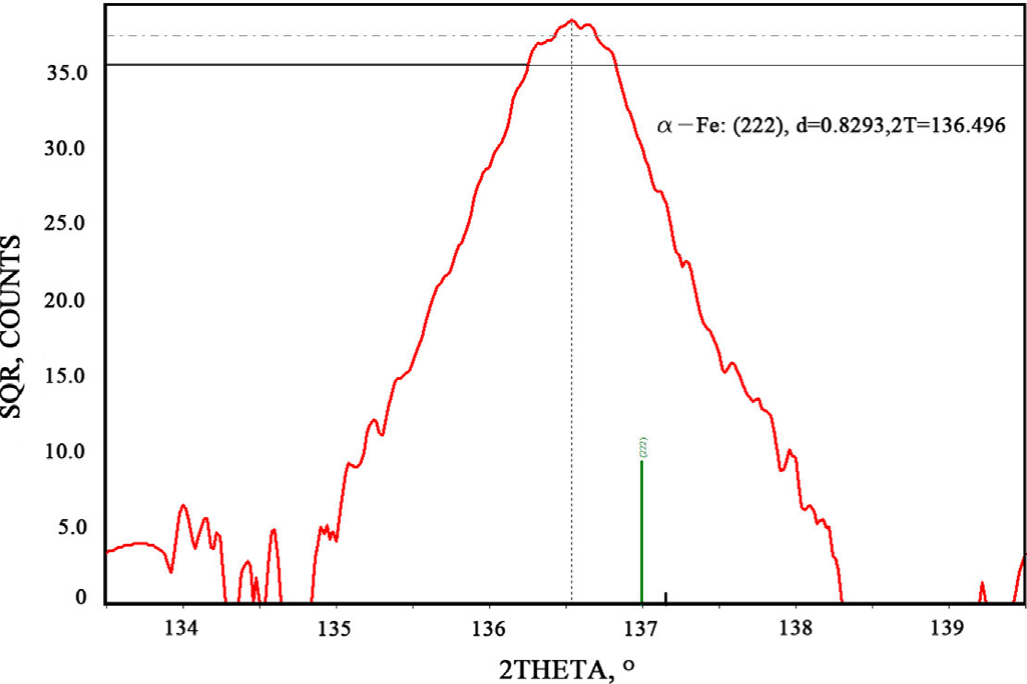
The energy spectrum of sample deformed at 940°C at (222) lattice plane after processed.

The spectrum of XRD gave results to determine the particle nature and the precipitation amount during deformation. However, the analysis of them should be very precise, since the slight change in the data would greatly affect the final results. Firstly, the spectrum of the specimen should be smoothed with parabolic filter of 13 points, and then justified by using the internal standard method (ISTD) with Si_2_O spectrum. After that, the spectrum should strip the background and K-alpha2 at the same time. When all the above steps were done, the phase search would be processed. The diffraction spectrum fitted well with the α-Fe energy spectrum. The spectrum result of D940 specimen, indicated with the value of diffraction lattice plane, the interplanar crystal spacing, and the 2theta are shown in Fig. 2. Since the closer the theta value approached 90, the more accurate the outcome became, the diffraction of lattice plane at (222) was chosen to be respective. The processed result is shown in Fig. 3.

## 4 Microstructure

**Fig. 4:**
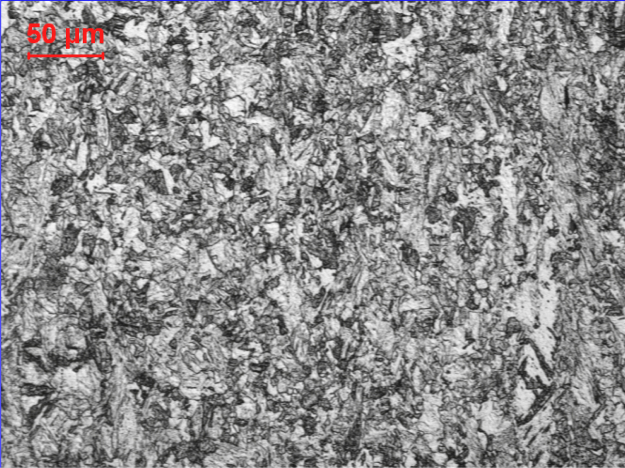
The microstructure of specimen that deformation at 900 °C.

The microstructure experienced metadynamic recrystallization (MDRX) when the steel was deformed at 900 °C, although no phase transformation took place, as indicated in Fig. 4. The MDRX governs the subsequent post-dynamic softening once the strain reaches the critical value [3,4]. Thus, the MDRX initials at 900 °C and refines the grains more effectively. However, since the temperature was not high enough for the grains to grow efficiently, the grains exhibited necklace-type morphology.
